# A Deep Neural Network-Based Model for Quantitative Evaluation of the Effects of Swimming Training

**DOI:** 10.1155/2022/5508365

**Published:** 2022-09-30

**Authors:** Jun-Jie Hou, Hui-Li Tian, Biao Lu

**Affiliations:** ^1^School of Physical Education, Suzhou University, Suzhou 234000, China; ^2^Philosophy of Sports, University of Perpetual Help System DALTA, Manila 0900, Philippines; ^3^Information Engineering Department, Suzhou University, Suzhou 234000, China

## Abstract

This paper analyzes the quantitative assessment model of the swimming training effect based on the deep neural network by constructing a deep neural network model and designing a quantitative assessment model of the swimming training effect. This paper addresses the problem of not considering the influence of the uncertainties existing in the virtual environment when evaluating swimming training and adds the power of the delays in the actual training operation environment, which is used to improve the objectivity and usability of swimming training evaluation results. To better measure the degree of influence of uncertainties, a training evaluation software module is developed to validate the usability of the simulated training evaluation method using simulated case data and compare it with the data after training evaluation using the unimproved evaluation method to verify the correctness and objectivity of the evaluation method in this paper. In the experiments, the feature extractor is a deep neural network, and the classifier is a gradient-boosting decision tree with integrated learning advantages. In the experimental comparison, we can achieve more than 60% accuracy and no more than a 1.00% decrease in recognition rate on DBPNN + GBDT, 78.5% parameter reduction, and 54.5% floating-point reduction on DPBNN. We can effectively reduce 32.1% of video memory occupation. It can be concluded from the experiments that deep neural network models are more effective and easier to obtain relatively accurate experimental results than shallow learning when facing high-dimensional sparse features. At the same time, deep neural networks can also improve the prediction results of external learning models. Therefore, the experimental results of this model are most intuitively accurate when combining deep neural networks with gradient boosting decision trees.

## 1. Introduction

Swimming and fitness are mass sports that are gaining more attention in the general environment of national fitness. Competitive swimming is the first sport to be officially included in the Olympic Games. At the present stage, the teaching and training of competitive swimming are still mainly based on education by example, that is, through the demonstration of the coach's movements and the self-correction of the students to achieve teaching and training [1]. The teaching model is not real time, the student's observation of the coach's standard movements is not comprehensive, and it is difficult for the coach to judge the correctness of the students' body movements scientifically, so the training efficiency is low. It enables athletes to conduct strength training and posture simulation on land, and students and coaches can observe and communicate technical movements in real time [2]. However, when athletes use swimming simulator training, the correctness of their sports posture still relies on the coach's manual judgment, and there is a lack of natural swimming sensation in the visual and physical aspects. The above status quo requires competitive swimming training to introduce advanced technology to scientifically analyze and optimize athletes' technical movements and provide a complete real swimming experience to keep athletes' training motivated.

Deep learning has become an important research direction of machine learning in recent years. Compared with traditional machine learning algorithms, deep learning algorithms do not need to go through feature engineering to obtain good enough data features, its deep structure can complete the learning process of components by itself, and there are enough trainable parameters, which makes deep learning able to train many samples with high accuracy [3]. Convolutional neural network (CNN) is one of the representative algorithms of deep understanding, whose basic structure consists of a convolutional layer, pooling layer, activation layer, and fully connected layer. Finally, the probability of each category is calculated by the fully connected layer and the classifier to realize the recognition of images [4]. The complete convolutional neural network is based on the CNN with the removal of the fully connected layer, using the deconvolution to enlarge the reduced feature map, recovering the detailed information by fusing the feature map obtained from the convolutional layer, and finally calculating the probability of each pixel belonging to each category on the feature map recovered to the input size to realize the semantic segmentation of the image [5]. Although deep neural networks have higher accuracy, they require a much larger number of labeled samples as support, and the training cost is enormous. Production of remote sensing image datasets involves a series of operations, such as correcting remote sensing images and then cropping and manual labeling, which consumes a lot of time and labor. And the use of small datasets for training the network is prone to over-fitting or under-fitting.

Deep learning methods have achieved good performance in many application domains. Still, until now, people have not been able to theoretically explain its effectiveness and consider it a black box model. On the other hand, the design and application of deep neural network structures are in urgent need of solid theories to guide them, but none of them has been accepted by most researchers yet. Despite the strong model representation capability of deep neural networks, the training optimization of deep neural network models is generally considered difficult [6]. The training optimization problem of deep neural networks is a high-dimensional nonconvex optimization problem, which is very different from the traditional machine learning optimization and the general mathematically studied nonconvex optimization problems [7]. Deep neural network training optimization is characterized by large scale, extensive data, and high-dimensional nonconvexity. Practical deep neural network models are often over-parameterized, which is very different from traditional machine learning optimization. Deep neural network models are large in scale, even tens of millions of dimensions, and mathematically studied nonconvex optimization theories generally do not address such high dimensions [8]. Note that the training optimization problem of deep neural networks includes many related issues, not only referring to optimization algorithm design and convergence analysis. In this paper, the relationship between swimming training and deep neural networks is explored to identify the connection between the two to determine the effectiveness of swimming training [9]. It is investigated using comparative experiments to analyze the influence of swimming training methods on the training effect. According to the research of this paper, it is beneficial to guide swimming teaching activities and provide technical reference for swimming coaches and has important practical significance for the development of swimming activities making full use of the sensitive period of physical quality development of swimmers as a window of opportunity for physical quality development, combine with the characteristics of swimming sport itself, introduce the concept of functional movement training in the process of physical training of swimmers, consolidate the material foundation, and optimize the training content. Through the modular design of training content, the physical training of swimmers can be scientific, systematic, and sustainable.

## 2. Related Works

Swimming training research gradually formed with the emergence of competitive swimming in the world competition in the late 1950s, which made a bare theoretical pavement for the development of swimming and made significant contributions to the development of swimming [10]. In recent years, academic research on swimming training has stagnated and developed during the development of swimming. Swimming training is in continuous product and improvement, both in theoretical research and practical activities [11]. Therefore, many experts, scholars, and front-line coaches are interested in exploring this field. The historical evolution of the hotspots of swimming training research as the direction of this study is a rational choice based on the unique charm of swimming training research and my strong interest in swimming training research.

The integration between disciplines is influenced by the development of information science and technology, and many scientific and technological developments also affect the outcome of swimming training. Still, the overall effect is always on the path of scientific, systematic, and comprehensive. The study attempts to summarize the development path and laws of current swimming training research and form a periodic theoretical system to provide an academic reference for future swimming training research [12]. At the same time, theory guides practice, and the theoretical development of swimming training has important guiding significance for swimming training activities. Based on the evolution law of the hotspots of swimming training theory and practice research, the future development direction of swimming training research is inferred by combining the characteristic properties of the swimming sport itself, the content system of swimming training, and the structural characteristics of swimming training [13, 14]. At the same time, to improve the training level of swimming training practice activities, it is necessary to master the theoretical development of swimming training. Gao et al. and Zhang et al. studied how swimming improved the nation's quality and mentioned that the water environment in which swimming exercise occurs is very different from the environment in which other types of exercise occur. Swimming, as a unique and attractive sport, not only has muscular fitness and fitness but also has the function of exercising people's will quality compared with other sports [15, 16]. Gao concluded from two months of swimming exercise that swimming could effectively change the body shape and various functions of college students under certain intensity [15]. Pawan et al. and Dewangan and Sahu tell us that swimming has specific fitness effects for different social groups by talking about the impact of swimming on human health [17, 18].

Deep neural networks (DNNs) have become one of the most popular research directions due to the excellence of deep learning methods, which is a further enhancement of artificial neural networks (ANNs) in the field of machine learning [19, 20]. This paper demonstrates that a neural network with multiple implicit layers can learn features better and obtain better results. Still, the learned features must have great typicality to help visualize and classify data clearly [21, 22]. Since then, the status of BP neural networks has been high, and many experts and researchers have taken deep learning methods as one of the leading research objects. Universities have also set up special research institutes to investigate deep neural networks' characteristics, trial scenarios, and advantages and disadvantages. Up to now, the theoretical research about deep BP neural networks is still in its initial stage except for the corresponding explanation from the bionic perspective. Still, it has achieved excellent results in many applications to perform deep learning of functions [23, 24]. At the same time, its use in image recognition is relatively early and more mature; Lin et al. explains referring to a deep neural network with a convolutional structure inspired by a biological vision model called a convolutional neural network [25]. In an image recognition competition, Hinton and his student Alex Krzyzewski and others used CNNs that benefited from rich data and improved GPU computing power to get the best competitive results. Since then, BP network models have been widely used in graphic images to improve accuracy and reduce the timelines involved in human eigenvalue processing, resulting in a convenient way of image recognition [26, 27].

## 3. Quantitative Evaluation Model Design of Swimming Training Effect Based on Depth Neural Network

### 3.1. Depth Neural Network Model Construction

**Figure 1:**
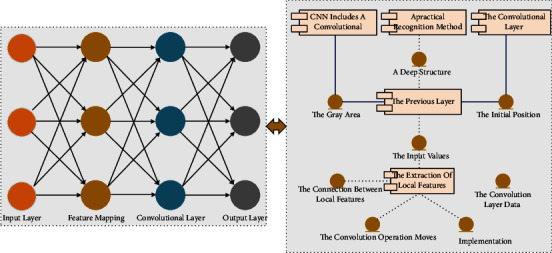
Deep convolutional neural network structure.

Deep convolutional neural network CNN includes a convolutional layer and pooling layer, a practical recognition method that has become popular in recent years. As shown in Figure 1, the convolutional layer neural network contains two layers: one is the feature extraction layer, and the other is the feature mapping layer. The feature extraction layer includes the convolutional layer and the pooling layer, its unique network layer. The input layer of each neuron is connected to the local domain of the previous layer, which can complete the extraction of local features, and the connection between local features and other features is fixed after extraction. A feature mapping layer is a two-dimensional plane; the neurons on each aircraft can share the weights, and the input data and the convolution layer data are set by themselves; when the convolution operation moves one position at a time, the actual process of its implementation is to complete the first convolution from the initial position, and the convolution kernel and the gray area of the input values in the graph are multiplied bit by bit and then summed up; that is, × 1  +  0  ×  2  +  1 ×  3 + 0 × 2 + 1 × 3 + 0 × 4 + 1 × 3 + 0 × 4 + 1 ×  5 = 15, 15 is the first value in the matrix after the convolution operation. The main difference is the different approach to layer definition and depth processing in multilayer perceptron. Deep neural networks mimic how the human brain thinks by first building single-layer neurons layer by layer so that one single-layer network is trained at a time. After all, layers are trained, and a wake-sleep algorithm is used for tuning. Convolutional neural networks are mediated by “convolutional kernels.” The same convolution kernel is shared across all images, and the image retains its original position relationship after the convolution operation.

A CNN is a feed-forward neural network with a deep structure that contains many convolutional computational layers, which can provide an end-to-end approach to task analysis. CNN structures typically have multiple sets of learnable parameters and components. These components usually consist of an alternating cascade of four essential network layers: a convolutional layer, a pooling layer, an activation layer, and a regularization layer.

#### 3.1.1. Convolution Layer

The convolution layer further samples the output feature map of the previous layer, so generally, the size of the feature map everywhere will be smaller than the input feature map. To keep the size of the input and output feature maps constant, adding “0” to the edge of the feature map during the convolution process is necessary as a padding compensation operation. In addition, the convolution operation is a convolution kernel sliding step that is not a pixel-by-pixel wagon, sometimes skipping a few pixels, where the sliding width is called stride. Suppose here the input sample data of a three-dimensional tensor *I*_in_∈RC_in_ × *w*_in_ × *h*_in_, the convolution operation padding is *p*, and stride size is *s*, using a filter of the convolution layer, which is also a 3D tensor *F*_in_ ∈ RK × *K* × *C*_in_ × *C*_out_, assuming that the length and width of the convolution kernel are *K*, and the width and height of the output feature map after the convolution operation for this sample are

(1) $$\begin{array}{c} w_{\text{out}}=\sum^{}\frac{w_{\text{in}}+k-2p}{\sqrt{s-K}},\\ h_{\text{out}}=\sum^{}\frac{\sqrt{h_{\text{in}}-k+2p}}{s-K}. \end{array}$$

The output feature data and the input are three-dimensional tensors, which can be expressed as *I*_out_ ∈ RC_out_ × *W*_out_. Here, we use a sample feature map to illustrate that the convolutional neural network is trained with input data, which can significantly accelerate the training efficiency and converge more quickly. Usually, the amount of data for one training is batch, and the selection of batch depends on the sample size and memory conditions, so the input data is a four-dimensional tensor, and the first dimension is the value of the batch.

#### 3.1.2. Pooling Layer

The pooling layer, also known as the sink layer, aims to aggregate the data in the local perceptual field of the input feature map into a target value, which is designed to reduce the complexity of the network model and avoid overfitting with too many features. The pooling layer is like the convolutional layer in that it uses a convolutional kernel to extract the significant features of a perceptual field region. The only difference with the convolutional kernel of the convolutional layer is that the kernel parameter values are fixed here, and there is no need to use the backpropagation algorithm to update the parameters. Therefore, only the type of pooling layer (average pooling or maximum pooling), the size of the pooling kernel, and the pooling step are required for the training of the neural network, and the average pooling kernel extracts the average and maximum values of the local perceptual field area, respectively. It is defined as

(2) $$\begin{array}{c} f_{\left(n+1\right)}=\sum^{}\text{pool}\left(f_{j}^{n-1}+j\right). \end{array}$$

#### 3.1.3. Fully Connected Layers

Fully connected layers are generally in the last few layers in modern convolutional neural networks and play the role of feature classification in the whole network. The function of convolutional and pooling layers is to map the input feature representation to the circumscribed feature space. In contrast, fully connected layers map the circumscribed feature representation to the target label space. Earlier, multilayer perceptrons (fully connected networks) consisting of only fully connected layers were frequently used in some simple classification tasks, building a linear mapping of feature transformations. Still, such networks have poor nonlinear capabilities and insufficient processing power under complex tasks, so such networks are now used only in some low-end embedded devices for simple classification tasks.

The data processing process of the fully connected layer is essentially a process of a linear transformation of matrices to vectors, where the parameters that need to be updated using the backpropagation algorithm include the weight parameter matrix and the bias parameter vector. Since the input data of the fully connected layer are vectorized features, assuming that the single sample input feature is *x*∈*R*^*m*^, the number of output neurons (feature vector length) is *n*, the weight parameter matrix of the fully connected layer is *a*∈*Rn*^*∗*^*m*, and the bias parameter vector is *b*∈*Rn*, and then, the output eigenvector can be expressed as a linear matrix transformation as

(3) $$\begin{array}{c} {\text{feature}}_{\left(\text{out}-1\right)}=\sum^{}\frac{a\times x-b}{\sqrt{ax+b}}. \end{array}$$

From another perspective, each scalar value of the fully connected layer parameter matrix can be regarded as a convolutional kernel of size 1 × 1. The fully connected layer can be defined as a convolutional layer when running the network model. For the fully connected layer, the convolutional kernel size can be set to 1 × 1. For the fully connected layer after the convolutional layer, the convolutional kernel size can be defined as a global convolution of *h* × *w* where *h* and *w* are the height and width of the input feature map, respectively. The fully connected layer is usually not very computationally intensive. Still, the number of parameters is very high in the whole network model, and the number of parameters in the fully connected layer can reach more than 95% in the Alex Net and VGG-16 network models. AlexNet is an earlier deep network applied on ImageNet, and its accuracy greatly improved compared to traditional methods. Framework: 5 convolutional layers followed by 3 fully connected layers with ReLU activation function. One improvement of VGG16 over AlexNet is using several consecutive 3 × 3 convolutional kernels instead of the larger convolutional kernels (11 × 11, 5 × 5) in AlexNet. For a given perceptual field (the local size of the input picture for the output), the use of stacked small convolutional kernels is preferable to the use of large convolutional kernels because the multilayer nonlinear layers can increase the network depth to ensure learning more complex patterns, and at a more negligible cost. Still, the computation is less than 10%. With the continuous development of deep learning theory, modern deep neural networks gradually eliminate connected layers or use global average pooling instead of fully connected layers so that the number of layers in the network can be deepened while maintaining a relatively small number of parameters, reducing the training difficulty of the network and the risk of overfitting.

#### 3.1.4. Batch Normalization Layer

Batch normalization can speed up the model's convergence, solve the inherent problem of “gradient dispersion” in deep networks to a certain extent, and avoid the problem of complex parameter updates during backpropagation. In addition, batch normalization applies to deep networks and can play a good role in improving the generalization of traditional shallow simple network models. The batch normalization layer has become essential to almost all neural network models. The specific algorithmic process of batch normalization is mainly divided into four steps; first, for each batch sample data, calculate its overall sample mean and sample variance, assuming that the input batch data are *x* = (*x*^(1)^, *x*^(2)^, ...,*x*^(*m*)^), which is a feature matrix with sample size *m*, the statistical characteristics of the batch data.

(4) $$\begin{array}{c} ub=\int_{i=1}\frac{m+x^{i}}{m-x},\\ \sigma b=\overset{m}{\underset{i=1}{\int }}\frac{{\left(x^{i}-ub\right)}^{2}}{x^{i}+ub}. \end{array}$$

After obtaining the batch samples' characteristic mean and characteristic variance, the sample data are batch normalized. The data can be input to the next layer of the network in the form of a standard normal distribution. Suppose the data distribution of each layer of the input network is inconsistent. In that case, the parameters of each layer will have to be adjusted to fit the new data distribution, which undoubtedly increases the difficulty of training the network, a problem known as internal covariate shift. Therefore, we normalize the data so that the input data distribution is the same for each layer.

(5) $$\begin{array}{c} x^{i}=\sum^{}x^{i-1}+\frac{ub}{\sqrt{\sigma b+e}}+\sqrt{\sigma b-e}. \end{array}$$

But simply normalizing each layer's features will change the data's original distribution and reduce the feature representation of the samples. The neural network is essentially designed to learn the actual distribution of the features. Finally, the normalized data need to be moderately cheapened to expect an approximate restoration of the distribution of the original features.

(6) $$\begin{array}{c} y^{i}=\sum^{}r^{x-i}-\beta +y. \end{array}$$

### 3.2. Quantitative Assessment Model Design of Swimming Training Effect

**Figure 2:**
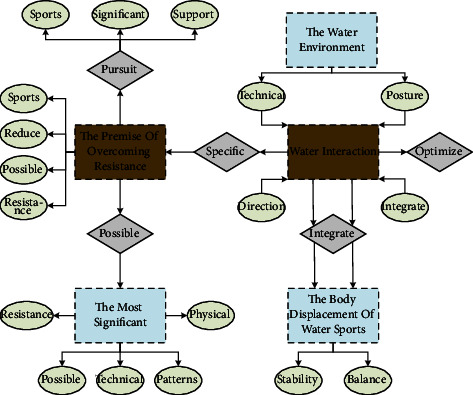
Swimming training process.

Swimming is a kind of sport in the water environment, with the premise of overcoming resistance in the water, using limbs and water interaction, and generating propulsive force to make the body displacement of water sports. Thismobile environment is the most significant and fundamental difference between swimming and other land sports, that is, there is no fixed support in the water. Because the environment is very different, many principles and laws applicable to land human sports cannot be applied to swimming and its unique hydrodynamic principle. That is to reduce the resistance as much as possible while increasing the propulsion resistance, so that as many people as possible can swim in the water. However, due to the difference in physical strength and skills, the degree of pursuing the two is as different as possible. The swimming training process is shown in Figure 2.

Specialized learning of technical movement patterns in the water is required to find relative stability and balance in the water and to exercise the coordination of the neuromuscular system specific to movement in the water, i.e., the same direction, when performed in the water and on land, the coordination between the neuro muscles will change because there is no buoyancy and water resistance on the ground. Therefore, swimmers need to closely integrate physical training with swimming techniques, optimize the power chain as much as possible while maintaining a stable streamlined body posture, and fully exploit and utilize the power of the muscles to generate maximum propulsion. This requires the athlete to have strong enough core area muscles to establish a stable support platform in the water, allowing for coordinated movement of the upper and lower extremities simultaneously to ensure that every part of the body functions efficiently. The aquatic environment in which swimming is located can prevent everyday communication and reduce the ability to regulate auditory and visual senses. Athletes are always relatively independent and need to give full play to their proprioceptive skills. It is difficult for coaches to provide real-time feedback to athletes during training or in the process of learning and improving swimming skills, which brings some hindrance to the formation of motor skills. The neuromuscular or proprioceptive system is very different in the water, so the change of muscle exertion in the water environment makes it much more difficult for athletes to handle the correct body posture in the water and achieve the movements required for competition.

**Figure 3:**
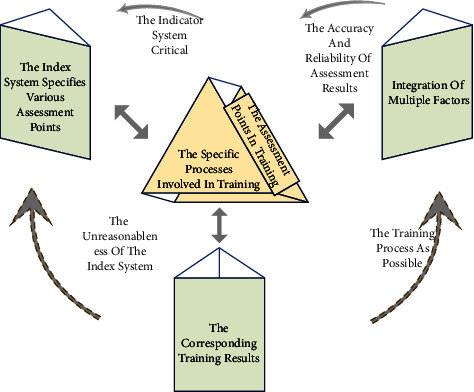
The construction process of the system of quantitative evaluation of swimming training.

The assessment indicator system plays a normative role as a guideline in training evaluation, making establishing the indicator system critical. In addition to using appropriate assessment methods, establishing a scientifically sound assessment index system is another crucial point in ensuring the accuracy and reliability of assessment results. This is because the index system specifies various assessment points, which are the abstraction and integration of multiple factors for the corresponding training results. One thing is sure; the so-called scientific and reasonable do not exist. To minimize the unreasonableness of the index system, it is necessary to achieve as complete coverage of the training process as possible. By abstracting the commonalities of the specific processes involved in training, through the process of dialectical logic, outline the fundamental features so that according to these features to complete the full coverage of the training process, then, the index system established by these features can be considered to some extent, and it is good coverage of all the assessment points in training. Because the content of the indicator system is highly specialized, in today's swimming training, the establishment of the indicator system often needs to first identify the specific type of swimming. After determining the swimming type, cooperate with relevant experts to ensure that the indicator system follows the principles of systematicness, objectivity, development, feasibility and comparability. According to the specific use and protection process of swimming, we analyze and abstract the elements involved in the training. Then, we will continue to discuss, classify, and screen in-depth, and finally determine the phased evaluation index system. The construction process of a quantitative assessment of swimming training is shown in Figure 3.

The assessment index system established through this process generally has comprehensive coverage and is very clear about the professional characteristics of swimming. The indexes continuously evaluated and screened by experts can better reflect the various skill points in using and securing this swimming. However, the attention to numerous details also leads to a more complex indicator system, and the increased complexity directly affects the complexity of solving the final assessment results. In addition, because of the great degree of attention to the details related to a particular swim, and the characteristics of different swims vary significantly from one another, it is necessary to reprofessionalize the analysis of other swims when establishing the index system; that is, it is required to develop other index systems for different swims. Still, it dramatically increases the difficulty of standardization and does not have generality. The training assessment system may be entirely different for other swims, which also increases the cost of training assessment. This project conducted relevant decision research to solve the above problems in the training evaluation system. The most prominent feature of the general index system in the establishment process is the abstraction of the training process according to the characteristics of a specific swim, which is the direct cause of the poor generality of the final system. Therefore, this paper considers changes in this point to make the established index system in line with the conception, in the abstraction of the swimming use and security process, not focus on the details and characteristics of each swim, but from a broad perspective to judge the training process.

The training was performed using supervised machine learning methods to provide a quantitative assessment of swimming functionality using three machine learning models: linear regression, support vector machine (SVM), and support vector regression (SVR), respectively. A more refined FNGS2.0 score was used to make the output more accurate regarding the label data and production of the pretrained models. The input vectors are [SymBrow, SymEye, SymMouth], and the output is the FNGS2.0 score. The pre-trained resulting model is used in the system to fit the quantitative data processed in real time. In this way, the system automatically and efficiently outputs the HBGS scores of swimming training by feeding the captured swimming training data in real time. Since our system is developed on a cell phone, athletes can assess at any time after swim training and obtain a real-time assessment of the swim training function.

## 4. Analysis of Results

### 4.1. Analysis of Quantitative Evaluation Model of Swimming Training Effect Based on Deep Neural Network

**Figure 4:**
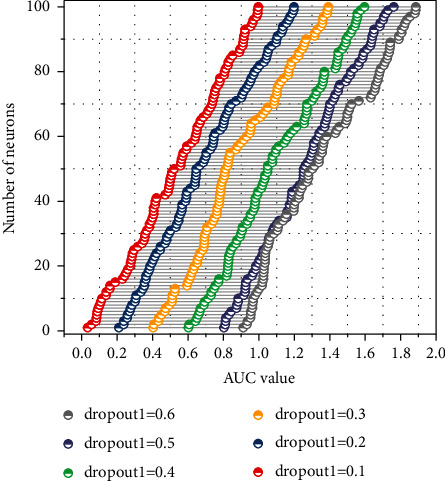
AUC values under the change of convolutional layer parameters.

A deep convolutional neural network contains several convolutional layers. To find the optimal network structure, the change in the number of neurons and the dropout ratio is analyzed by applying network search to the first convolutional layer (first defining the search space and the controller generating the network structure). The test results are recorded as shown in Figure 4. It can be observed that the dropout divergence trend is most apparent when there are 30 neurons, and the maximum AUC value in the test range appears at this time. Later, when the number of neurons was increased, the trend of Dropout0.4 and Dropout0.5 tended to coincide.

The optimal parameters of the first convolutional layer are obtained through the above experiments, and then, the other structural layers of the network perform the tuning operation; the number of neurons per layer is 10–100, the number of convolutional kernels is 2 or 6, the step size is 10, and the number of relatively optimal parameter units is 50. The number of convolutional kernels is 2. The convolutional neural network's convolutional kernels and pooling layers are changed using the adjusted parameters. Since the pooling layer in CNN has no parameters, dropout's critical role in the model is built. The pooling layer is not included in the model, and the dropout key role is in this model. The adjustment results are shown in Table 1.

To improve the algorithm accuracy, enhance the model generalization ability, and prevent overfitting, another factor affecting the model, regularization, is studied in-depth, and L2 regularization is added to the first layer of CNN in the three major feature extractors of NLP (by reducing the secondary dimensional weights in L2). As the value of L2 regularization increases from zero, the AUC values become unstable, and the model is poorly structured. The overfitting problem of the model is dealt with when dropout tuning is performed on the model, and there is no need to deal with it by L2 regularization.

**Figure 5:**
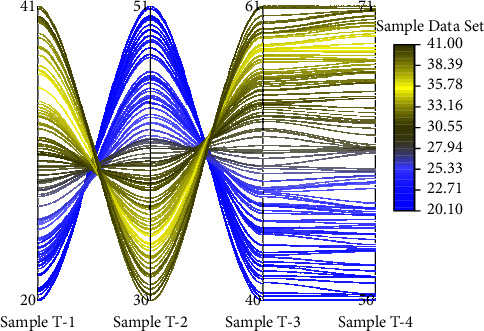
Swimming training effect data test.

The mathematical and statistical method focuses on the organization and statistical analysis of the experimental data, which will be counted in an Excel sheet. Programming support: SPSS supports rich data sources and has full data access and management capabilities as well as programming capabilities. Users only need to understand the principles of statistical analysis, and even if they are not familiar with the algorithms of statistical methods, they can quickly obtain the statistical analysis results they need, and it also supports secondary development. Powerful functions: SPSS has complete parts of data input, editing, statistical analysis, report and graph production, etc. It provides a comprehensive data analysis process and covers a full range of statistical analysis methods, such as exploratory analysis of data, partial correlation, analysis of variance, nonparametric test, multiple regression, logistic regression. And the collated data were analyzed using SPSS 20.0 with independent samples *t*-test. According to the experiment results, the mean ± standard was used to record and characterize the effects. At the same time, the data of some experimental items were organized and analyzed using *P*-value analysis to determine the correlation of the experimental results. The swimming training effect data test is shown in Figure 5.

Model evaluation is integral to the model development testing and validation process. It effectively discovers the optimal model for expressing the data and how well the model works. In this paper, a confusion matrix is chosen to evaluate the prediction results of the model. The confusion matrix is an intuitive presentation tool for evaluating classification models that the number of correct and incorrect predictions output by the classification model on the data, where each column of the matrix is a sample prediction of the model's forecast, and each row of the matrix is the sample reality.

**Figure 6:**
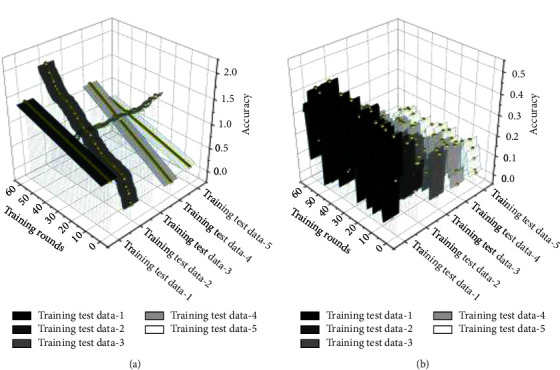
Comparison of model evaluation graphs. (a) Training rounds. (b) Training rounds.

The real-time data sending and receiving directly affects the immersion of the user's swimming experience; the shorter the delay, the lower the user's vertigo and the stronger the sense of reality. Two methods can test real time; one is to observe the smoothness of the field of view update through the user's experience and directly feel the size of the delay of the area of view update; the smoother the lot of view update, the lower the delay, the better the real time. However, this method lacks a quantitative description of the real-time performance. Another way calculates the frame rate by the number of frames of bit-pose data received by the virtual scene program in a fixed time. For Unity3D, the higher the scene refresh frame rate is, the smoother and more natural the motion display is, and a soft motion effect is achieved when the frame rate is above 30 fps. A comparison of the model evaluation graph is shown in Figure 6.

### 4.2. Quantitative Evaluation Model Implementation of Swimming Training Effect

The effectiveness of the proposed weight-based scaling invariant neural network integration method is verified through experiments on several datasets and various popular deep neural network models. A comparative analysis is performed with recently proposed deep neural network integration methods; the dropout value in the hidden layer is taken as 0.35, the number of neurons and other influencing parameters are taken as the optimal parameters tested in the past, and the input layer in the LR and GBDT models are taken as the features of the third hidden layer, and the dimensionality of the run to the features of the model is 50, which is 301 lower than the original dimensionality, and the specific dimensionality values are combined with linear function and nonlinear function, and the results are obtained after multiple layers of operations, which can be used as a reference standard to reduce the computational load of the model data.

**Figure 7:**
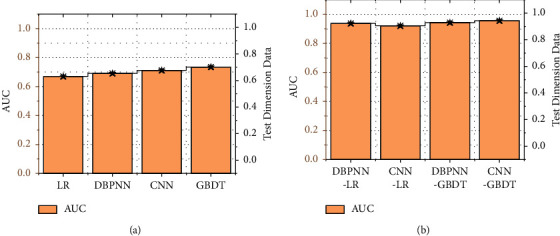
Comparison of AUC before and after model combination. (a) Before model combination. (b) After model combination.

In this CNN model, we choose 1D convolution Conv1D, which is only convolution in the width direction, and do not use a 2D array as the output representation, also known as feature map Conv2D convolution, the width of 1D convolution is 3, not 3 × 3 2D kernel array, 1D convolution is often used for sequence data, and the input of Conv1d is 3D data. Two-dimensional convolution is commonly used in computer vision and image processing. This model building does not involve feature map convolution, so the one-dimensional convolution Conv1D, which is suitable for this study, is used. Here, in LR and GBDT, input has been established with the upper layer link fully connected layer of features, observation can be seen that the new test dimension (80) only accounts for about 50% of the original size (351), and the features are extracted at the same time the dimensionality reduction is completed. The comparison of AUC before and after the model combination is shown in Figure 7.

**Figure 8:**
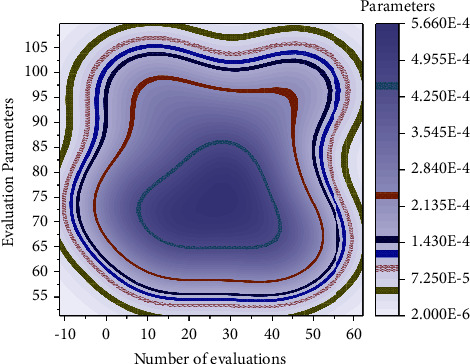
Comparison of training effect evaluation results.

The established combined model is debugged, and the obtained data are compared with the single model parameters. The gradient boosting decision tree generates *N* trees, and after receiving a sample message, each tree goes from the node of the root to the node of the leaf, and when it reaches the leaf node, it is 1 or 0, point or no point. The output value of each tree can be regarded as the output feature, taking the value of 0 or 1, a total of *N* trees, each tree *i* has Mi leaves can be seen as having *M* kinds of combinations, a tree corresponds to a one-hot encoding method, a total of multiple dimensions of new features, as the input vector into the LR model, the output of the result. The above operation is used to verify two things that the experimental results of the established deep learning combinations are generally better than the ordinary single model and to confirm that the deep learning models based on deep neural networks can improve the shallow learning methods of the single model. Collating the experimental results and comparing the metrics, it is observed that the combined models, such as (GBDT + DBPNN), are better than the general models, such as LR/GBDT/. It indicates that the collaborative models established in this study to enhance learning efficiency are effective and practical. During the experiment, it is relatively challenging to seek the optimal parameters of CNN, but its advantages as feature parameters are also apparent. The evaluation results obtained from several sets of training data by the evaluation method in this paper are compared with the evaluation results in the ideal training state without considering the uncertainties, and the corresponding practical operation results are shown in Figure 8.

The existing index system for training assessment is highly correlated with swimming training, making it necessary to establish a different index system adapted to current swimming for additional training due to the other characteristics and assessment concerns between their swimming training. To solve the abstraction problem to some extent, this paper proposes an assessment method decoupled from specific swims, blurring the guarantee and use characteristics of swim training and obtaining an upper-level index system after the abstraction of assessment points. In the implementation of the assessment, the lower layer can be based on the characteristics of the current swimming training with targeted training project assessment and the assessment of the results of the data obtained from the assessment of categorization and integration, such as the assessment of the use of a training multiple ways of operation together as the assessment of swimming training use operation results, the formation of the input data in line with the structure of the upper layer index system. Such an approach makes it unnecessary to establish separate index systems for assessing each swimming training in the bottom layer. Still, it also ensures the accuracy of the training assessment because the bottom layer is carried out according to the subdivision of different training. The general training assessment only considers the assessment of each technical point in the swimming protection training, ignoring the differences between the simulated training and the operational training in the virtual environment caused by external uncertainties, thus making the results of the mock training assessment may be somewhat different from the results of the actual operation. On the other hand, the use of hierarchical analysis can more clearly describe and quantify the factors in the training assessment, but dynamic changes in the uncertainty of elements cannot be directly quantified. Therefore, this paper proposes using deep neural networks for projection to assist the quantification process of the indeterminate factors, update the index system to match them, and finally get the assessment results closer to the actual operation level through the projection of hierarchical analysis.

## 5. Conclusion

The development of swimming training is the process of human beings in the continuous understanding of the laws of swimming movement and the physiological and psychological changes of human activity. The emergence of competitive swimming as a social phenomenon has pushed the curtain on swimming training. This paper studies the quantitative evaluation of the swimming training effect based on a deep neural network. DBPNN + GBDT is selected as the algorithm basis for model building, and swimming training test data are introduced for evaluation. The DPBNN used in this paper combines the advantages of the BP neural network and deep learning network algorithms, which enhances the model's massively parallel, distributed processing, self-organization, and self-learning capabilities and plays a significant role in performing also positive extraction and high-dimensional data processing in terms of automation degree, convolution, and sharing. After completing the deep neural network construction, the fusion gradient boosting decision tree model broadens the data types that can be processed to improve model prediction accuracy further. In this paper, based on the existing evaluation methods, the overall evaluation method and the corresponding index system are improved to establish a higher-level evaluation index system to shield the different characteristics of each equipment training, given the poor generality of the evaluation and the lack of consideration of the fundamental environmental factors in the evaluation results of the simulation training. At the same time, the effectiveness of the proposed evaluation method is verified by introducing a machine learning method that can obtain better data training results in small samples and dynamically obtain the influence weights of variable factors to achieve the coverage and consideration of external factors in the evaluation. The current initialization methods of deep neural networks are overwhelming. Most of them are symmetric random initialization, and the so-called symmetric random initialization means that all the parameters of the neural network ownership value are drawn from some distribution of density distribution about the origin, and the commonly used Gaussian random initialization is the symmetric random initialization. The standard Gaussian random initialization is symmetric random initialization. The activation functions of most of the popular deep network models are Relu functions because their activation regions are not symmetric. The activation for negative values of the input is 0.

## Figures and Tables

**Table 1:** Experimental results after adjusting the convolution kernel and pooling layer.

Parameter adjustment	Dropout	AUC
Optimal results	0.1763	0.7788
Convolution kernel = 1	0.1976	0.7695
Convolution kernel = 2	0.1884	0.7742
Convolution kernel = 3	0.1683	0.7681
Convolution kernel = 4	0.1739	0.7735
Convolution kernel = 5	0.197	0.7883
Convolution kernel = 6	0.1749	0.778
Convolution kernel = 7	0.1954	0.7748
Convolution kernel = 8	0.1722	0.7885

## Data Availability

The data used to support the findings of this study are available from the corresponding author upon request.
